# Supplementary material for: Cell Type-Specific Patterns in the Accumulation of DNA Damage Following Multifractional Radiation Exposure
Source: Int J Mol Sci. 2022 Oct 25;23(21):12861. doi: 10.3390/ijms232112861 (PMC9655477; doi:10.3390/ijms232112861)
Supplement: Supplementary file 1 [file ijms-23-12861-s001.zip › ijms-1892578-supplementary.pdf]

Supplementary figures

DFRE

VH10 (9 fractions)	B0	SEM	B1	SEM	B2	SEM
Control	0.021	0.617	0.633	0.142	-0.004	0.006
0.25Gy/fraction	0.022	0.575	0.561	0.132	-0.005	0.006
0.5 Gy/fraction	0.036	0.528	0.472	0.121	-0.007	0.006
1.0 Gy/fraction	0.050	0.508	0.421	0.117	-0.008	0.005
2.0 Gy/fraction	0.010	0.586	0.419	0.135	-0.011	0.006

DFRE

VH10 (12 fractions)	B0	SEM	B1	SEM	B2	SEM
Control	0.066	0.717	0.605	0.121	-0.003	0.004
0.25Gy/fraction	0.040	0.673	0.551	0.114	-0.005	0.004
0.5 Gy/fraction	0.142	0.612	0.407	0.104	-0.003	0.004
1.0 Gy/fraction	0.126	0.578	0.374	0.098	-0.006	0.003
2.0 Gy/fraction	0.102	0.591	0.362	0.100	-0.008	0.003

DFRE

AHH-1 (10 fractions)	B0	SEM	B1	SEM	B2	SEM
Control	0.095	0.193	0.859	0.074	0.019	0.006
0.25Gy/fraction	0.125	0.141	0.753	0.054	0.012	0.004
0.5 Gy/fraction	0.126	0,187	0.647	0.072	0.005	0.006
1.0 Gy/fraction	0.067	0.227	0.522	0.087	-0.017	0.007
2.0 Gy/fraction	0.045	0.405	0.305	0.156	-0.039	0.012

Table S1. Summary of B0, B1 and B2 coefficients from fitting the cell growth curves of VH10 fibroblasts (9 and 12 fractions) and AHH-1 lymphoblasts. DFRE during fractionated radiation exposure. Statistical analysis was carried out with GraphPad prism.

PFRE

VH10 (9 fractions)	B0	SEM	B1	SEM	B2	SEM
Control	0.04	0.26	0.326	0.04	0.003	0.001
0.25Gy/fraction	0.05	0.45	0.311	0.07	0.003	0.002
0.5 Gy/fraction	-0.01	0.40	0.298	0.06	0.002	0.002
1.0 Gy/fraction	0.02	0.55	0.132	0.09	0.002	0.003
2.0 Gy/fraction	0.16	0.88	0.070	0.14	0.005	0.005

PFRE

VH10 (12 fractions)	B0	SEM	B1	SEM	B2	SEM
Control	0.104	1.07	0.30	0.17	0.004	0.005
0.25Gy/fraction	0.004	0.71	0.28	0.11	0.004	0.004
0.5 Gy/fraction	-0.062	0.35	0.25	0.06	-0.001	0.002
1.0 Gy/fraction	-0.075	0.68	0.13	0.11	0.002	0.003
2.0 Gy/fraction	-0.111	0.68	-0.09	0.11	0.005	0.003

PFRE

AHH-1 (10 fractions)	B0	SEM	B1	SEM	B2	SEM
Control	0.07	0.237	1.080	0.06	0.002	0.003
0.25Gy/fraction	0.08	0.219	1.007	0.05	0.005	0.003
0.5 Gy/fraction	0.23	0.235	0.868	0.06	0.011	0.003
1.0 Gy/fraction	0.24	0.287	0.370	0.07	0.029	0.003
2.0 Gy/fraction	0.73	0.873	0.509	0.21	0.045	0.010

Table S2. Summary of B0, B1 and B2 coefficients from fitting the cell growth curves of VH10 fibroblasts (9 and 12 fractions) and AHH-1 lymphoblasts. PFRE post fractionated radiation exposure. Statistical analysis was carried out with GraphPad prism.

Cell growth

VH10 (9 fractions)	B0	SEM	B1	SEM	B2	SEM
Control	-0.04	0.670	0.682	0.06	-0.006	0.001
0.25Gy/fraction	-0.26	0.780	0.563	0.07	-0.005	0.001
0.5 Gy/fraction	0.00	0.720	0.391	0.07	-0.002	0.001
1.0 Gy/fraction	0.38	0.810	0.258	0.08	-0.001	0.001
2.0 Gy/fraction	0.75	1.140	0.161	0.11	-0.001	0.002

Cell growth

VH10 (12 fractions)	B0	SEM	B1	SEM	B2	SEM
Control	-0.071	1.23	0.67	0.10	-0.006	0.002
0.25Gy/fraction	0.157	0.95	0.53	0.08	-0.004	0.001
0.5 Gy/fraction	0.408	0.73	0.35	0.06	-0.002	0.001
1.0 Gy/fraction	0.748	0.92	0.22	0.08	-0.001	0.001
2.0 Gy/fraction	1.013	0.90	0.14	0.08	-0.001	0.001

Cell growth

AHH-1 (10 fractions)	B0	SEM	B1	SEM	B2	SEM
Control	-0.64	0.206	1.13	0.03	0.000	0.001
0.25Gy/fraction	-0.40	0.165	0.863	0.02	0.006	0.001
0.5 Gy/fraction	-0.04	0.218	0.567	0.03	0.012	0.001
1.0 Gy/fraction	1.01	0.367	-0.026	0.05	0.021	0.002
2.0 Gy/fraction	1.92	0.748	-0.576	0.11	0.021	0.003

Table S3. Summary of B0, B1 and B2 coefficients from fitting the cell growth curves of VH10 fibroblasts (9 and 12 fractions) and AHH-1 lymphoblasts. PFRE post fractionated radiation exposure. Statistical analysis was carried out with GraphPad prism.

VH10 fibroblasts (9 fractions)			
**DFRE**	**95% confidence interval**	**Adjusted p-Value**	**Significance**
Control vs 0.25 Gy/fraction	-0.5307 to 0.6741	0.99	ns
Control vs 0.5 Gy/fraction	-0.4414 to 0.7634	0.90	ns
Control vs 1.0 Gy/fraction	-0.3905 to 0.8143	0.7739	ns
Control vs 2.0 Gy/fraction	-0.3887 to 0.8161	0.7688	ns
0.25 Gy/fraction vs 0.5 Gy/fraction	-0.5131 to 0.6917	0.9868	ns
0.25 Gy/fraction vs 1.0 Gy/fraction	-0.4622 to 0.7426	0.9348	ns
0.25 Gy/fraction vs 2.0 Gy/fraction	-0.4604 to 0.7444	0.932	ns
0.5 Gy/fraction vs 1.0 Gy/fraction	-0.5515 to 0.6533	0.9985	ns
0.5 Gy/fraction vs 2.0 Gy/fraction	-0.5497 to 0.6551	0.9982	ns
1.0 Gy/fraction vs 2.0 Gy/fraction	-0.6006 to 0.6042	>0.9999	ns
**PFRE**	**95% confidence interval**	**Adjusted p-Value**	**Significance**
Control vs 0.25 Gy/fraction	-0.3963 to 0.4269	>0.9999	ns
Control vs 0.5 Gy/fraction	-0.3834 to 0.4398	0.9993	ns
Control vs 1.0 Gy/fraction	-0.2182 to 0.6050	0.5586	ns
Control vs 2.0 Gy/fraction	-0.01147 to 0.8118	0.0576	ns
0.25 Gy/fraction vs 0.5 Gy/fraction	-0.3987 to 0.4245	>0.9999	ns
0.25 Gy/fraction vs 1.0 Gy/fraction	-0.2335 to 0.5897	0.6275	ns
0.25 Gy/fraction vs 2.0 Gy/fraction	-0.02677 to 0.7965	0.0697	ns
0.5 Gy/fraction vs 1.0 Gy/fraction	-0.2464 to 0.5768	0.6857	ns
0.5 Gy/fraction vs 2.0 Gy/fraction	-0.03967 to 0.7836	0.0816	ns
1.0 Gy/fraction vs 2.0 Gy/fraction	-0.2049 to 0.6184	0.5	ns
**CELL GROWTH**	**95% confidence interval**	**Adjusted p-Value**	**Significance**
Control vs 0.25 Gy/fraction	-0.2565 to 0.4943	0.8306	ns
Control vs 0.5 Gy/fraction	-0.08405 to 0.6668	0.1537	ns
Control vs 1.0 Gy/fraction	0.04835 to 0.7992	0.0259	*
Control vs 2.0 Gy/fraction	0.1458 to 0.8966	0.0071	**
0.25 Gy/fraction vs 0.5 Gy/fraction	-0.2029 to 0.5479	0.5778	ns
0.25 Gy/fraction vs 1.0 Gy/fraction	-0.07055 to 0.6803	0.1289	ns
0.25 Gy/fraction vs 2.0 Gy/fraction	0.02685 to 0.7777	0.0347	*
0.5 Gy/fraction vs 1.0 Gy/fraction	-0.2430 to 0.5078	0.7724	ns
0.5 Gy/fraction vs 2.0 Gy/fraction	-0.1456 to 0.6052	0.3256	ns
1.0 Gy/fraction vs 2.0 Gy/fraction	-0.2780 to 0.4728	0.9072	ns

Table S4. Summary of one-way ANOVA of the B1 coefficients cell growth curves of VH10 fibroblasts (9 fractions). DFRE during radiation exposure, PFRE post fractionated radiation exposure. Statistical analysis was carried out with GraphPad prism. * represent p values <0.05, ** represents p values <0.01.

VH10 fibroblasts (12 fractions)			
**DFRE**	**95% confidence interval**	**Adjusted p-Value**	**Significance**
Control vs 0.25 Gy/fraction	-0.4467 to 0.5557	1.00	ns
Control vs 0.5 Gy/fraction	-0.3027 to 0.6997	0.70	ns
Control vs 1.0 Gy/fraction	-0.2697 to 0.7327	0.5732	ns
Control vs 2.0 Gy/fraction	-0.2583 to 0.7441	0.5316	ns
0.25 Gy/fraction vs 0.5 Gy/fraction	-0.3572 to 0.6452	0.8727	ns
0.25 Gy/fraction vs 1.0 Gy/fraction	-0.3242 to 0.6782	0.7715	ns
0.25 Gy/fraction vs 2.0 Gy/fraction	-0.3128 to 0.6896	0.7318	ns
0.5 Gy/fraction vs 1.0 Gy/fraction	-0.4682 to 0.5342	0.9994	ns
0.5 Gy/fraction vs 2.0 Gy/fraction	-0.4568 to 0.5456	0.9981	ns
1.0 Gy/fraction vs 2.0 Gy/fraction	-0.4898 to 0.5126	>0.9999	ns
**PFRE**	**95% confidence interval**	**Adjusted p-Value**	**Significance**
Control vs 0.25 Gy/fraction	-0.5274 to 0.5690	>0.9999	ns
Control vs 0.5 Gy/fraction	-0.4914 to 0.6050	0.9966	ns
Control vs 1.0 Gy/fraction	-0.3792 to 0.7172	0.8431	ns
Control vs 2.0 Gy/fraction	-0.1540 to 0.9423	0.2018	ns
0.25 Gy/fraction vs 0.5 Gy/fraction	-0.5122 to 0.5842	0.9994	ns
0.25 Gy/fraction vs 1.0 Gy/fraction	-0.4000 to 0.6964	0.8944	ns
0.25 Gy/fraction vs 2.0 Gy/fraction	-0.1748 to 0.9215	0.2404	ns
0.5 Gy/fraction vs 1.0 Gy/fraction	-0.4360 to 0.6604	0.9578	ns
0.5 Gy/fraction vs 2.0 Gy/fraction	-0.2108 to 0.8855	0.3211	ns
1.0 Gy/fraction vs 2.0 Gy/fraction	-0.3230 to 0.7733	0.6685	ns
**CELL GROWTH**	**95% confidence interval**	**Adjusted p-Value**	**Significance**
Control vs 0.25 Gy/fraction	-0.2438 to 0.5160	0.7628	ns
Control vs 0.5 Gy/fraction	-0.05926 to 0.7006	0.1101	ns
Control vs 1.0 Gy/fraction	0.07255 to 0.8324	0.0189	*
Control vs 2.0 Gy/fraction	0.1538 to 0.9137	0.0065	**
0.25 Gy/fraction vs 0.5 Gy/fraction	-0.1954 to 0.5645	0.5296	ns
0.25 Gy/fraction vs 1.0 Gy/fraction	-0.06357 to 0.6963	0.1165	ns
0.25 Gy/fraction vs 2.0 Gy/fraction	0.01773 to 0.7776	0.0394	*
0.5 Gy/fraction vs 1.0 Gy/fraction	-0.2481 to 0.5117	0.782	ns
0.5 Gy/fraction vs 2.0 Gy/fraction	-0.1668 to 0.5930	0.4014	ns
1.0 Gy/fraction vs 2.0 Gy/fraction	-0.2986 to 0.4612	0.9509	ns

Table S5. Summary of one-way ANOVA on cell growth curves of B1 coefficients VH10 fibroblasts (12 fractions). DFRE during radiation exposure, PFRE post fractionated radiation exposure. Statistical analysis was carried out with GraphPad prism. * represent p values <0.05, ** represents p values <0.01.

AHH-1 Lymphoblasts (10 fractions)			
**DFRE**	**95% confidence interval**	**Adjusted p-Value**	**Significance**
Control vs 0.25 Gy/fraction	-0.4889 to 0.2967	0.94	ns
Control vs 0.5 Gy/fraction	-0.5307 to 0.2549	0.81	ns
Control vs 1.0 Gy/fraction	-0.7431 to 0.04252	0.092	ns
Control vs 2.0 Gy/fraction	-1.035 to -0.2496	0.0012	**
0.25Gy/fraction vs 0.5 Gy/fraction	-0.4346 to 0.3510	0.9972	ns
0.25Gy/fraction vs 1.0 Gy/fraction	-0.6470 to 0.1386	0.313	ns
0.25Gy/fraction vs 2.0 Gy/fraction	-0.9391 to -0.1535	0.0049	**
0.5Gy/fraction vs 1.0 Gy/fraction	-0.6052 to 0.1804	0.4797	ns
0.5Gy/fraction vs 2.0 Gy/fraction	-0.8973 to -0.1117	0.0093	**
1.00Gy/fraction vs 2.0 Gy/fraction	-0.6849 to 0.1007	0.1996	ns
**PFRE**	**95% confidence interval**	**Adjusted p-Value**	**Significance**
Control vs 0.25 Gy/fraction	-0.1603 to 0.3063	0.9878	ns
Control vs 0.5 Gy/fraction	-0.02133 to 0.4453	0.6352	ns
Control vs 1.0 Gy/fraction	0.4766 to 0.9432	0.0023	**
Control vs 2.0 Gy/fraction	1.355 to 1.822	<0.0001	****
0.25 Gy/fraction vs 0.5Gy/fraction	-0.09433 to 0.3723	0.8849	ns
0.25 Gy/fraction vs 1.0Gy/fraction	0.4036 to 0.8702	0.0057	**
0.25 Gy/fraction vs 2.0Gy/fraction	1.282 to 1.749	<0.0001	****
0.5 Gy/fraction vs 1.00 Gy/fraction	0.2646 to 0.7312	0.0339	*
0.5 Gy/fraction vs 2.0 Gy/fraction	1.143 to 1.610	<0.0001	****
1.0 Gy/fraction vs 2.0 Gy/fraction	0.6455 to 1.112	0.0003	***
**CELL GROWTH**	**95% confidence interval**	**Adjusted p-Value**	**Significance**
Control vs 0.25 Gy/fraction	0.01001 to 0.5234	0.0399	*
Control vs 0.5 Gy/fraction	0.3061 to 0.8195	<0.0001	****
Control vs 1.0 Gy/fraction	0.8993 to 1.413	<0.0001	****
Control vs 2.0 Gy/fraction	1.450 to 1.963	<0.0001	****
0.25Gy/fraction vs 0.5 Gy/fraction	0.03941 to 0.5528	0.0203	*
0.25Gy/fraction vs 1.0 Gy/fraction	0.6326 to 1.146	<0.0001	****
0.25Gy/fraction vs 2.0 Gy/fraction	1.183 to 1.696	<0.0001	****
0.5Gy/fraction vs 1.0 Gy/fraction	0.3365 to 0.8498	<0.0001	****
0.5Gy/fraction vs 2.0 Gy/fraction	0.8867 to 1.400	<0.0001	****
1.00Gy/fraction vs 2.0 Gy/fraction	0.2936 to 0.8069	<0.0001	****

Table S6. Summary of one-way ANOVA on cell growth curves of B1 coefficients AHH-1 lymphoblasts (10 fractions). DFRE during radiation exposure, PFRE post fractionated radiation

exposure. Statistical analysis was carried out with GraphPad prism. * represent p values <0.05, ** represents p values <0.01, *** represents p values <0.001, **** represents p values <0.0001

**FOCI**	**3 Days PFRE**			
**Total foci (TF)**	**α**	**STD**	**p-value (chi squared test)**	**(R^2 test)**
VH10 (9 fractions)	0.21	0.18	0.028	0.94
VH10 (12 fractions)	0.038	0.020	0.041	0.63
AHH-1 (10 fractions)	0.067	0.029	0.589	0.84
**Small foci (SF)**	**α**	**STD**	**p-value (chi squared test)**	**(R^2 test)**
VH10 (9 fractions)	0.20	0.17	0.047	0.95
VH10 (12 fractions)	0.023	0.014	0	0.60
AHH-1 (10 fractions)	0.053	0.035	0.516	0.86
**Large foci (LF)**	**α**	**STD**	**p-value (chi squared test)**	**(R^2 test)**
VH10 (9 fractions)	0.0219	0.0035	0.273	0.92
VH10 (12 fractions)	0.0027	0.0011	0.094	0.72
AHH-1 (10 fractions)	-0.0006	0.0033	0.278	0.66

Table S7. Summary of α coefficients from linear dose response relationship of foci data three days post fractionated radiation. STD represents ± standard deviations. Statistical analysis was carried out with Python 3 software.

	7 Days PFRE							
**Total foci TF**	**B0**	**STD**	**B1**	**STD**	**B2**	**STD**	**p-value (chi squared test)**	**(R^2 test)**
VH10 (9 fractions)	0	0	-0.74	0.22	0.0603	0.015	0.186	0.89
VH10 (12 fractions)	0	0	0.0093	0.0091	0.0021	0.0011	0.114	0.97
AHH-1 (10 fractions)	0	0	-0.057	0.019	0.0014	0.0010	0.048	0.66
**Small foci SF**	**B0**	**STD**	**B1**	**STD**	**B2**	**STD**	**p-value (chi squared test)**	**(R^2 test)**
VH10 (9 fractions)	0	0	-0.70	0.21	0.057	0.015	0.192	0.88
VH10 (12 fractions)	0	0	0.0076	0.0093	0.00223	0.00097	0.118	0.96
AHH-1 (10 fractions)	0	0	-0.053	0.019	0.0010	0.0011	0.063	0.67
**Large foci LF**	**B0**	**STD**	**B1**	**STD**	**B2**	**STD**	**p-value (chi squared test)**	**(R^2 test)**
VH10 (9 fractions)	0	0	-0.0258	0.0031	0.00220	0.00024	0.009	0.99
VH10 (12 fractions)	0	0	0.0005	0.0013	0.00016	0.00014	0.236	0.96
AHH-1 (10 fractions)	0	0	-0.0029	0.0036	0.00027	0.00022	0.095	0.47

Table S8. Summary of B0, B1, and B2 coefficients from linear quadratic dose response relationship of foci data seven days post fractionated radiation. STD represents ± standard deviations. Statistical analysis was carried out with Python 3 software.

	3 Days PFRE						
**Micronuclei (MN)**	**a**	**b**	**c**	**u (a)**	**u(b)**	**u(c)**	**R^2**
VH10 (9 fractions)	-0.063	2.221	1.783	0.016	0.293	0.529	0.96
VH10 (12 fractions)	-0.036	1.781	-0.279	0.032	0.733	3.206	0.97
AHH-1 (10 fractions)	0.026	0.124	1.027	0.015	0.176	0.349	0.99
	**7 Days PFRE**						
**Micronuclei (MN)**	**a**	**b**	**c**	**u (a)**	**u(b)**	**u(c)**	**R^2**
VH10 (9 fractions)	0.914	0.631	0.000	0.123	0.098	0.000	0.98
VH10 (12 fractions)	0.845	1.404	0.000	0.188	0.365	0.000	0.98
AHH-1 (10 fractions)	0.026	0.109	0.337	0.021	0.117	0.133	0.96

Table S9. a, b and c coefficients from fitting MN frequency in both VH10 fibroblasts (after 9, and 12 fractions) and AHH-1 lymphoblasts, to a linear quadratic function 3 and 7 days PFRE. Statistical analysis was carried out with Python.

	3 Days PFRE				
**Nuclear buds (NBD)**	**a**	**b**	**u (a)**	**u(b)**	**R^2**
VH10 (9 fractions)	0.510	0.331	0.112	0.139	0.960
VH10 (12 fractions)	0.237	0.214	0.063	0.153	0.990
	**7 Days PFRE**				
**Nuclear buds (NBD)**	**a**	**b**	**u (a)**	**u(b)**	**R^2**
VH10 (9 fractions)	0.534	0.440	0.230	0.219	0.940
VH10 (12 fractions)	0.308	0.147	0.062	0.166	0.990

	3 Days PFRE				
**Nuclear buds (NBD)**	**a**	**b**	**u (a)**	**u(b)**	**R2**
**VH10 (9 fractions)**	0,510	0,331	0,112	0,139	0,960
**VH10 (12 fractions)**	0,237	0,214	0,063	0,153	0,990
	**7 Days PFRE**				
**Nuclear buds (NBD)**	**a**	**b**	**u (a)**	**u(b)**	**R2**

**VH10 (9 fractions)**	0,534	0,440	0,230	0,219	0,940
**VH10 (12 fractions)**	0,308	0,147	0,062	0,166	0,990

Table S10. a, and b coefficients from fitting NBD frequency in VH10 fibroblasts (after 9, and 12 fractions) to a linear function 3 and 7 days PFRE. Statistical analysis was carried out with Python.

	**3 Days PFRE**						
**Giant mononucleus (GN)**	**a**	**b**	**c**	**u (a)**	**u(b)**	**u(c)**	**R^2**
**AHH-1 (10 fractions)**	0.018	0.146	0.364	0.020	0.122	0.134	0.94
	**7 Days PFRE**						
**Giant mononucleus (GN)**	**a**	**b**	**c**	**u (a)**	**u(b)**	**u(c)**	**R^2**
**AHH-1 (10 fractions)**	0.025	0.047	0.428	0.017	0.194	0.395	0.99

Table S11. a, b, and c coefficients from fitting GN frequency in AHH-1 lymphoblasts to a linear quadratic function 3 and 7 days PFRE. Statistical analysis was carried out with Python.

**Colony formation**	**Coefficients**		**Standard deviation**		**R^2 value**
	**α**	**β**	**α**	**β**	
VH10 (9 fractions)	0.005	0.0043	0.021	0.0019	0.6030
VH10 (12 fractions)	-0.027	0.0033	0.042	0.0028	0.4545
AHH-1 (10 fractions)	-0.084	0.0066	0.02	0.0016	0.6646

Table S12. α and β coefficients from linear quadratic $S = e^{-(D(\alpha+\beta d))}$data fitting for colony formation 10 days post fractionated radiation exposure

VH10 fibroblasts (9 fractions)	Coefficients		Standard error		R^2 values
	**α**	**β**	**α**	**β**	
Control	0.295	0.022	0.056	0.016	0.9865
0.25 Gy/fraction	-0.002	0.080	0.054	0.018	0.9847
0.5 Gy/fraction	0.284	0.023	0.124	0.035	0.9370
1.0 Gy/fraction	0.162	0.047	0.048	0.015	0.9895

Table S13. α and β coefficients from linear quadratic data fitting $S = e^{-(\alpha D+\beta D^2)}$ for radiosensitivity with clonogenic cell survival 20 days after radiation exposure. Survival curves for VH10 fibroblast was fit to linear quadratic equation, while AHH-1 survival curves were fit to a linear equation.

AHH-1 lymphoblasts (10 fractions)	Coefficients	Standard error	R^2 values
	**α**	**α**	
Control	-0.3611	0.02449	0.9666
0.25Gy/fraction	-0.3267	0.01884	0.9733
0.5 Gy/fraction	-0.3033	0.01961	0.9644
1.0 Gy/fraction	-0.2622	0.01426	0.9725
2.0 Gy/fraction	-0.2547	0.02627	0.9065

Table S14. α coefficients from linear data fitting $S = e^{-(\alpha D)}$ for the survival curves of AHH-1 20 days post fractionated radiation exposure
